# Association between thyroid function and 10-year cardiovascular disease risk of patients with diabetes: a cross-sectional study using KNHANES 2013–2014

**DOI:** 10.12701/jyms.2026.43.28

**Published:** 2026-04-08

**Authors:** Bogyeong Kim, Minjin Jeon, Yuseop Lee, Hyunji Reem, Seung Min Chung

**Affiliations:** 1 Yeungnam University College of Medicine, Daegu, Korea; 2 Division of Endocrinology and Metabolism, Department of Internal Medicine, Yeungnam University College of Medicine, Daegu, Korea

**Keywords:** Cardiovascular disease risk, Diabetes mellitus, Glycemic control, Hypothyroidism, Korea National Health and Nutrition Examination Survey, Thyrotropin

## Abstract

**Background:**

This study investigated the association between thyroid function and the 10-year cardiovascular disease (CVD) risk in patients with diabetes.

**Methods:**

This retrospective cross-sectional study analyzed 246 patients with diabetes aged 30 to 79 years (weighted n=1,611,708) from the 2013 to 2014 Korea National Health and Nutrition Examination Survey. Individuals with a history of CVD, thyroid disease, or pregnancy were excluded. Thyroid function was categorized as euthyroid, hypothyroid, or hyperthyroid, based on serum thyroid-stimulating hormone (TSH) levels (reference in Koreans, 0.62–6.86 mIU/L). The 10-year CVD risk was estimated using the Predicting Risk of CVD EVENTs calculator.

**Results:**

The mean participant age was 54.3±10.1 years, with a male-to-female ratio of 1.41:1. Although patients with hypothyroid (14.1%) and hyperthyroid (15.7%) statuses showed a higher CVD risk than those with euthyroid status (12.3%), the difference was not statistically significant (*p*=0.337). However, in the hypothyroid group (n=11), TSH levels showed a moderate correlation with 10-year CVD risk (r=0.603, *p*<0.05), although this finding should be interpreted with caution owing to the small sample size. After adjusting for diabetes- and thyroid-related variables, TSH level was independently associated with higher estimated 10-year CVD risk, particularly in patients with a glycated hemoglobin level of <7% (adjusted coefficient, 0.17; 95% confidence interval, 0.05–0.29; *p*<0.05).

**Conclusion:**

Monitoring TSH levels in patients with well-controlled diabetes may provide additional information regarding the risk of CVD; however, prospective studies are needed to confirm the long-term prognostic value.

## Introduction

Diabetes is a globally prevalent metabolic disorder that affects individuals irrespective of sex, race, and age. In 2021, approximately 529 million people were diagnosed with diabetes globally, with an age-standardized prevalence rate of approximately 6.1% [1]. In South Korea, approximately 12.5% of adults aged ≥19 years and over 28% of those aged ≥60 years were diagnosed with diabetes in 2022; this finding highlights the growing health concern within the country [2].

Diabetes is strongly associated with an increased risk of cardiovascular disease (CVD) including coronary artery disease (CAD), stroke, heart failure (HF), and peripheral artery disease. Patients with diabetes have a higher prevalence of CVD than healthy individuals, indicating that diabetes independently contributes to an increased risk of CVD and its mortality [3-5]. Therefore, the early identification and prevention of cardiovascular risk factors in patients with diabetes is crucial.

Thyroid hormones maintain cardiovascular homeostasis, and thyroid dysfunction is strongly associated with an increased incidence of CAD and cardiovascular mortality [6-9]. Several studies have indicated that thyroid dysfunction (hyperthyroidism or hypothyroidism) is associated with an increased risk of CVD and mortality in the general population [10-14]. However, studies have shown conflicting results regarding this association among patients with diabetes [15-17]. Therefore, future studies should identify specific groups in which thyroid dysfunction affects the CVD risk in patients with diabetes.

Therefore, we aimed to evaluate the effect of thyroid function on the 10-year CVD risk using the Predicting Risk of Cardiovascular Disease EVENTs (PREVENT) calculator [18] in Korean subjects with diabetes nationwide. We also aimed to determine the subgroups in which thyroid dysfunction significantly affected the 10-year CVD risk.

## Methods

**Ethics statement:** This study was based on publicly available and fully anonymized data from the Korea National Health and Nutrition Examination Survey (KNHANES; accessed November 2024). Ethical approval was granted by the Institutional Review Board (IRB) of Yeungnam University (IRB No: 7002016-E-2024-132), and the requirement for informed consent was waived.

### 1. Study population

**Fig. 1.**
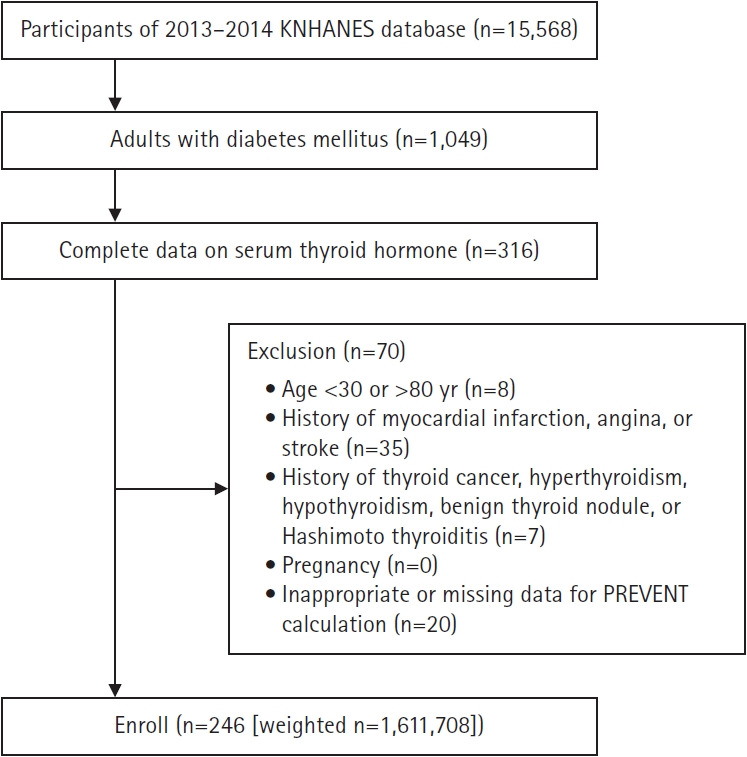
Study population. KNHANES, Korea National Health and Nutrition Examination Survey; PREVENT, Predicting Risk of Cardiovascular Disease EVENTs.

In this cross-sectional study, data were obtained from the KNHANES between January 1, 2013 and December 31, 2014. The data were obtained from a third-party source (i.e., KNHANES) and, therefore, cannot be made publicly available by the authors. However, the datasets are publicly accessible through the KNHANES website (https://knhanes.kdca.go.kr/knhanes/main.do) subject to the policies of the data provider. KNHANES is a cross-sectional population-based nationwide survey conducted regularly by the Korea Centers for Disease Control and Prevention [19]. Of 15,568 participants, 1,049 with diabetes were initially selected. Among them, 316 individuals with available thyroid hormone blood test results were included in the study. The exclusion criteria were as follows: age <30 or >80 years (n=8); history of myocardial infarction, angina, or stroke (n=35); history of thyroid disorders, including thyroid cancer, hyperthyroidism, hypothyroidism, benign thyroid nodules, or Hashimoto’s thyroiditis (n=7); current pregnancy (n=0); and missing essential data or values outside the applicable range for the PREVENT calculator (n=20). After the exclusion of 70 individuals, 246 participants were included in the final retrospective analysis (Fig. 1).

### 2. Data collection

The following patient characteristics were obtained: sex; age; height; body mass index (BMI); waist circumference; systolic and diastolic blood pressure (BP); smoking and drinking status; engagement in regular exercise; medical history of hypertension, dyslipidemia, and diabetes; and laboratory profiles. BMI was calculated as body weight divided by height squared (kg/m^2^). Systolic and diastolic BP were calculated by averaging the second and third measurements. Smoking status was classified as ever- or never-smoker, or current smoker. Drinking status was categorized as drinker and nondrinker. Regular exercise was defined as walking for ≥30 minutes 5 days per week, performing moderate-intensity physical activity for ≥2 hours 30 min/week, performing high-intensity physical activity for ≥1 hour 15 min/week, or performing combined moderate- and high-intensity physical activity (1 minute at high intensity=2 minutes at moderate intensity).

Diabetes was defined according to one of the following criteria: fasting blood glucose level ≥126 mg/dL (measured after a minimum of 8 hours of fasting), prior physician diagnosis, or current use of oral hypoglycemic agents or insulin therapy. Diabetes duration was calculated by subtracting the age at diagnosis from the patient’s current age. For patients without a prior diagnosis by a physician, the duration of diabetes was recorded as 0 years. A glycated hemoglobin (HbA1c) level of <7% was defined as well-controlled blood glucose [20]. Patients currently using antihypertensive and lipid-lowering agents were classified as having hypertension and dyslipidemia, respectively.

Venous blood samples were collected after fasting for 8 hours. Blood test results, including HbA1c, fasting glucose, total cholesterol, high-density lipoprotein (HDL) cholesterol, triglycerides (TG), creatinine, thyroid-stimulating hormone (TSH), free thyroxine (fT4), and antithyroid peroxidase (TPO) antibody levels, were collected. Additionally, urinary creatinine, albumin, and iodine levels were measured. Low-density lipoprotein cholesterol (LDL-C) levels were calculated using the Friedewald equation: LDL-C=total cholesterol−HDL cholesterol−(TG/5). The estimated glomerular filtration rate (eGFR) was determined using the modification of diet in renal disease equation: eGFR=175×(serum creatinine)^−1.154^×(age)^−0.203^×0.742 (if female). The urinary albumin-creatinine ratio (uACR) was calculated using the following equation, recommended by the American Diabetes Association [21]: uACR (mg/g)=urine albumin concentration (mg/L)/urine creatinine concentration (mg/dL). The urinary iodine/creatinine ratio (UIC) was measured as follows [22]: UIC (μg/g)=urine iodide concentration (μg/L)/urine creatinine concentration (g/L).

### 3. Assessment of thyroid function

Thyroid function status was categorized according to the reference range established by the 2013 to 2015 KNHANES, as specified by the Korean Endocrine Society [23]. The TSH and fT4 reference ranges were 0.62 to 6.86 mIU/L and 0.9 to 1.8 ng/dL, respectively. Patients were classified as having hypothyroidism (TSH ≥6.86 mIU/L), hyperthyroidism (TSH <0.62 mIU/L), or euthyroidism (TSH and fT4 within the reference range).

### 4. Risk of cardiovascular disease determined using the PREVENT online calculator

The PREVENT online calculator, developed by the American Heart Association (AHA) Cardiovascular–Kidney–Metabolic Scientific Advisory Group, is designed for healthcare professionals and individuals to estimate 10-year CVD risk. It is based on data from more than 6 million patient cases and guidelines from the AHA and the American College of Cardiology. It incorporates established models such as the Framingham Risk Score and the atherosclerotic cardiovascular disease (ASCVD) risk calculator. The risk calculation applies to primary prevention for patients aged 30 to 79 years without a history of CAD, stroke, or HF. The essential risk factors incorporated into the calculation include age, sex, total cholesterol level, HDL cholesterol level, systolic BP, BMI, eGFR, diabetes status, current smoking status, and use of antihypertensive or lipid-lowering medications. Additionally, optional predictors include uACR, HbA1c level, and social deprivation index at the ZIP Code level. In this study, uACR and HbA1c levels were used to estimate the 10-year CVD risk. In accordance with the AHA guidelines, patients were categorized as high and low-to-intermediate risk if their estimated 10-year CVD risk was ≥20% and <20%, respectively [24]. The 10-year risks of ASCVD and HF were also calculated.

### 5. Statistical analysis

The data were statistically analyzed using IBM SPSS ver. 27 (IBM Corp., Armonk, NY, USA) and R version 4.4.0 (R Foundation for Statistical Computing, Vienna, Austria). All the analyses were conducted using a complex sample design framework that incorporated stratification (kstrata), clustering (psu), and weights (wt_itvex). A complex sample general linear model procedure was used. Continuous variables are expressed as weighted means±standard deviations, while categorical variables are presented as unweighted frequencies and weighted percentages (n [%]); *p*-values were derived from the complex sample analyses. The correlations between variables were assessed within the complex sampling framework. The relationship between thyroid function and 10-year CVD risk was analyzed using a linear regression procedure. Subgroup analyses stratified by glycemic control (HbA1c <7% vs. ≥7%) were performed. Statistical significance was set at *p*<0.05.

## Results

### 1. Baseline characteristics

The mean age of the 246 participants (weighted n=1,611,708) was 54.3±10.1 years, with a male-to-female ratio of 1.41:1. The participants were categorized into low-to-intermediate (n=198 [weighted n=1,352,396]) and high (n=48 [weighted n=259,312]) 10-year CVD risk groups. The baseline characteristics of each group are summarized in Supplementary Table 1. The high-risk group was significantly older and had a higher prevalence of hypertension and a longer duration of diabetes; and exhibited poorer systolic BP, HbA1c, creatinine, eGFR, and uACR levels than the low-to-intermediate risk group did. However, thyroid hormone levels did not differ significantly between the two groups.

The baseline characteristics according to euthyroid (n=230 [weighted n=1,492,669]), hypothyroid (n=11 [weighted n=81,030]), and hyperthyroid (n=5 [weighted n=38,008]) statuses are summarized in Table 1. The three groups showed no significant differences in age or sex, but their drinking history, diabetes duration, and UIC (*p*<0.05) varied significantly. The proportion of drinkers in the hyperthyroid group was lower and the duration of diabetes was shorter than that in the other groups. The uACR and UIC were the highest in the euthyroid and hypothyroid groups, respectively.

**Fig. 2.**
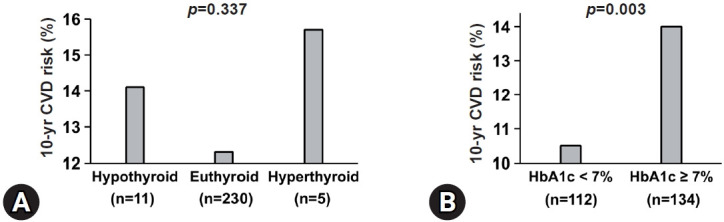
Ten-year cardiovascular disease (CVD) risk score according to (A) thyroid function status and (B) glycated hemoglobin (HbA1c) levels.

The 10-year CVD risks were 12.3%±8.9%, 14.1%±6.6%, and 15.7%±6.8% in the euthyroid, hypothyroid, and hyperthyroid groups, respectively, but their differences were not significant (*p*=0.337) (Fig. 2A). The 10-year CVD risk of individuals with poor glycemic control (HbA1c ≥7%) was significantly higher (14.0%±9.9%) than that of individuals with good glycemic control (HbA1c <7%, 10.5%±6.8%; *p*=0.003) (Fig. 2B).

### 2. Correlation between thyroid function and 10-year cardiovascular disease risk score according to thyroid function status

The correlations between TSH level, fT4 level, and 10-year CVD risk in the euthyroid, hypothyroid, and hyperthyroid groups are summarized in Table 2. TSH and fT4 levels and 10-year CVD risk scores were not significantly correlated in the euthyroid and hyperthyroid groups. However, TSH level exhibited a significant positive correlation with 10-year CVD, ASCVD, and HF risk in the hypothyroid group (weighted Pearson correlation r=0.603, 0.608, and 0.653, respectively; *p*<0.05). Additionally, fT4 level demonstrated a significant negative correlation with 10-year HF risk (r=−0.608, *p*<0.05). Because the hypothyroid (n=11) and hyperthyroid (n=5) subgroups had small sample sizes, the estimates may have been less stable and should, therefore, be interpreted with caution.

### 3. Effect of thyroid function on the increased 10-year cardiovascular disease risk

Linear regression analysis was conducted to examine whether TSH and fT4 levels contributed to the increased 10-year CVD risk in patients with diabetes (Table 3). Since metabolic and lifestyle-related factors were adequately reflected in the 10-year CVD risk calculation formula, diabetes-related (HbA1c and diabetes duration) and thyroid-related (anti-TPO antibody and UIC) variables were selected as covariates and adjusted. After multivariable adjustment, TSH and fT4 levels were not identified as independent determinants of the overall population’s increased 10-year CVD, ASCVD, or HF risk. A subgroup analysis based on HbA1c levels (<7% vs. ≥7%) was performed. This subgroup analysis was conducted as an exploratory analysis to investigate the potential effects of modification based on glycemic control status. Interestingly, in the good-glycemic-control subgroup (HbA1c <7%), TSH levels were independently associated with higher estimated 10-year CVD (adjusted coefficient, 0.17; 95% confidence interval [CI], 0.05–0.29; *p*=0.005), ASCVD (adjusted coefficient, 0.1; 95% CI, 0.02–0.18; *p*=0.014), and HF (adjusted coefficient, 0.12; 95% CI, 0.05–0.19; *p*=0.001) risks.

Linear regression analysis was performed to investigate whether thyroid function status influenced the increased 10-year CVD risk (Supplementary Table 2). The same covariates listed in Table 3 were considered. Thyroid function status was not a significant predictor of high 10-year CVD risk in the overall population or in the sub-analysis stratified by HbA1c level (<7% vs. ≥7%).

## Discussion

This study aimed to evaluate the clinical utility of thyroid function tests in predicting the 10-year CVD risk in patients with diabetes. Among patients with diabetes from the 2013 to 2014 KNHANES (weighted n=1,611,708), the hypothyroid and hyperthyroid groups had higher 10-year CVD risk scores than the euthyroid group did; however, the differences were not statistically significant. The glycemic control status (HbA1c <7% vs. ≥7%) showed a significant association with differences in 10-year CVD risk scores. Although increased TSH levels were not significantly related to increased 10-year CVD risk in the overall diabetic population, higher TSH levels were significantly associated with increased 10-year CVD, ASCVD, and HF risk in the subgroup with HbA1c levels of <7%. In the hypothyroid group, TSH levels also showed a significant positive correlation with 10-year CVD, ASCVD, and HF risks. However, these findings should be interpreted as hypothesis-generating owing to the exploratory nature of the subgroup analyses, particularly given the small sample size of the hypothyroid group (n=11).

Given the high prevalence of CVD and its adverse prognosis in patients with diabetes [3-5], early identification of predictive markers and risk factors is essential for the effective prevention and management of CVD. The PREVENT calculator estimates predicted 10- and 30-year CVD risks in adults aged 30 to 79 years without established CVD. It is designed to address the limitations of the Pooled Cohort Equations, which tend to overestimate risk, and to expand the applicable age range for risk assessment. Notably, the model accounts for previously unaddressed factors and enhances equity across diverse populations by eliminating race as a variable. Therefore, the PREVENT calculator can be used to predict CVD risk more precisely and identify individuals at high risk. Consequently, a comprehensive prevention strategy can be developed [18,25]

Thyroid dysfunction is more common in patients with diabetes (type 1 or type 2) than in the general population; therefore, it may increase the risk of metabolic disorders and CVD [26,27]. In patients with diabetes, this condition is linked to dyslipidemia, insulin resistance, and endothelial dysfunction, all of which contribute to an increased CVD risk [26]. Even within the euthyroid range, low and high TSH levels are associated with increased all-cause and CVD mortality [28]. Therefore, thyroid function in patients with diabetes should be assessed and managed as part of a comprehensive strategy to reduce cardiovascular complications [27].

In this study, TSH levels were positively correlated with 10-year CVD, ASCVD, and HF risks in patients with diabetes and hypothyroidism. Previous studies have examined the relationship between thyroid function and CVD in patients with diabetes and hypothyroidism; however, the findings have been inconsistent. Some studies have shown that subclinical or overt hypothyroidism in patients with type 2 diabetes is associated with coronary heart disease in Chinese and Ghanaian populations [16,29]. Conversely, studies in the United Kingdom and Australia have indicated that subclinical hypothyroidism does not increase CVD events or mortality in patients with type 2 diabetes [30,31]. These discrepancies may be attributed to variations in the study populations, demographic characteristics, and research methodology. Therefore, well-designed prospective cohort studies should be conducted to explore the potential relationship between thyroid function and CVD risk in patients with diabetes.

Previous prospective cohort studies have indicated that in addition to lifestyle factors (i.e., smoking, alcohol consumption, physical activity, and diet quality) and the presence of metabolic syndrome, HbA1c level is an important contributor to CVD risk in patients with type 2 diabetes [32,33]. The present study found that the 10-year CVD risk in individuals with poor glycemic control (HbA1c ≥7%) was significantly higher than that in individuals with good glycemic control (HbA1c <7%). Notably, TSH levels were independently associated with 10-year CVD, ASCVD, and HF risks within the subgroup maintaining good glycemic control. However, this association was not observed in individuals with poor glycemic control. Therefore, although the management of HbA1c is crucial for reducing 10-year CVD risk in patients with diabetes, monitoring TSH levels in individuals with well-controlled diabetes may enhance the assessment of 10-year CVD risk.

This study has several strengths. First, although the sample size was relatively small (n=246), survey weights were applied to improve the representativeness of the Korean population. Second, thyroid function status was categorized according to the Korean-specific TSH reference range, which was higher than that in Western countries [23]. However, this study has some limitations. First, the cross-sectional nature of the study may not reflect changes in thyroid function over time, making it difficult to interpret causality. Second, medical history based on questionnaires might have been subject to selection bias [34]. Third, the limited number of participants in the hypothyroid (n=11) and hyperthyroid (n=5) groups limited our ability to classify subclinical thyroid dysfunction as an independent statistical category. Accordingly, subgroup analyses, including those involving thyroid dysfunction and those stratified by glycemic control, should be interpreted as exploratory and hypothesis-generating, and the possibility of limited statistical power and multiplicity due to multiple subgroup analyses should be considered. Fourth, only TSH and fT4 levels were determined, and the lack of triiodothyronine (T3) measurements limited a more comprehensive physiological interpretation of the thyroid-CVD axis. Regarding the association between low free T3 levels and increased risk of CVD mortality in subjects with diabetes [17,35], various thyroid hormone levels should be measured in future studies. Lastly, because the PREVENT calculator estimates predicted CVD events rather than observed events, the risks estimated in this study should be interpreted as indicators of relative associations within the cohort rather than absolute risk predictions. Furthermore, because the PREVENT calculator was developed using United States-based cohorts and incorporates a social deprivation index derived from United States ZIP Codes, it may not fully reflect Korea’s socioeconomic context.

In conclusion, increased TSH levels in patients with diabetes correlated with an increased 10-year risk of CVD, particularly in patients with diabetes and hypothyroidism. TSH levels may provide additional prognostic information regarding the estimated 10-year CVD risk in patients with well-controlled diabetes (i.e., HbA1c levels of <7%).

## Figures and Tables

**Table 1.** Baseline characteristics based on thyroid function status

Characteristic	Total	Thyroid function status			*p*-value
		Euthyroid	Hypothyroid	Hyperthyroid	
No. of patients	246 (100)	230 (93.5)	11 (4.5)	5 (2.0)	
Sex					
Male	144 (63.9)	134 (63.5)	6 (67.5)	4 (75.7)	0.844
Female	102 (36.1)	96 (36.5)	5 (32.5)	1 (24.3)	
Age (yr)	54.3±10.1	54.2±10.3	55.6±6.6	58.2±8.5	0.487
Smoking					
Former or never	175 (66.4)	163 (66.7)	8 (58.0)	4 (72.4)	0.857
Current	71 (33.6)	67 (33.3)	3 (42.0)	1 (27.6)	
Drinking					
Never	29 (10.7)	26 (10.0)	1 (4.0)	2 (51.9)	0.004
Yes	217 (89.3)	204 (90.0)	10 (96.0)	3 (48.1)	
Regular exercise	119 (48.4)	115 (50.4)	3 (29.5)	1 (10.8)	0.103
Hypertension	109 (37.7)	101 (37.7)	6 (41.4)	2 (31.2)	0.957
Dyslipidemia	60 (22.5)	57 (22.8)	3 (27.5)	0 (0)	0.616
Duration of diabetes (yr)	4.5±6.0	4.5±6.1	4.7±5.2	0.8±1.1	<0.001
Body mass index (kg/m^2^)	25.8±3.3	25.8±3.3	25.0±4.2	27.1±2.5	0.391
Waist circumference (cm)	87.6±8.6	87.7±8.6	85.3±8.4	92.0±9.2	0.371
Systolic BP (mmHg)	123.5±15.6	123.1±15.1	131.0±23.1	119.6±13.9	0.515
Diastolic BP (mmHg)	78.1±10.8	78.0±10.5	81.8±13.7	75.1±16.0	0.676
Fasting glucose (mg/dL)	143.2±37.7	142.9±37.6	142.9±36.5	158.8±49.0	0.750
HbA1c (%)	7.4±1.5	7.4±1.5	7.5±1.4	7.9±1.6	0.710
Total cholesterol (mg/dL)	191.6±36.1	191.3±36.3	191.4±36.3	203.3±32.5	0.693
HDL-cholesterol (mg/dL)	45.6±10.4	46.0±10.3	41.5±10.3	41.8±11.6	0.115
LDL-cholesterol (mg/dL)	105.6±36.3	105.6±36.4	104.2±39.9	108.3±34.6	0.977
Triglycerides (mg/dL)	201.6±148.0	198.5±144.0	228.7±211.1	266.0±166.1	0.582
Creatinine (mg/dL)	0.9±0.3	0.9±0.3	1.0±0.3	1.2±0.7	0.561
eGFR (mL/min/1.73 m^2^)	83.8±18.5	84.2±18.2	79.7±19.5	75.2±27.4	0.569
UACR (mg/g)	35.5±109.9	37.2±114.0	14.4±21.4	15.1±8.1	0.051
UIC (μg/g)	537.1±1195.4	536.5±1219.5	718.8±982.5	265.1±227.0	0.047
TSH (mIU/L)	3.2±5.8	2.4±1.2	19.6±19.7	0.5±0.2	<0.001
Free thyroxine (ng/dL)	1.2±0.2	1.2±0.2	1.1±0.3	1.4±0.3	0.030
Anti-TPO antibody (IU/mL)	32.3±165.8	19.9±115.8	272.7±511.4	9.3±3.5	0.163

Values are presented as unweighted frequencies (n) with weighted percentages (%) for categorical variables and mean±standard deviation for continuous variables. All estimates were calculated by accounting for the complex survey design, including stratification, clustering, and sampling weights. BP, blood pressure; HDL, high-density lipoprotein; LDL, low-density lipoprotein; eGFR, estimated glomerular filtration rate; UACR, urinary albumin-creatinine ratio; UIC, urinary iodine/creatinine ratio; TSH, thyroid-stimulating hormone; TPO, thyroid peroxidase.

**Table 2.** Correlation analysis between TSH, fT4, and 10-year CVD risk score according to thyroid function status

Thyroid status	No. of patients	CVD risk	ASCVD risk	HF risk
Euthyroid	230			
TSH		–0.009	–0.049	–0.016
fT4		0.097	0.107	0.097
Hypothyroid	11			
TSH		0.603^a)^	0.608^a)^	0.653^a)^
fT4		–0.572	0.535	–0.608^a)^
Hyperthyroid	5			
TSH		–0.260	–0.264	–0.080
fT4		0.051	0.053	–0.161

Numbers correspond to Pearson correlation r within the complex sampling framework. TSH, thyroid-stimulating hormone; fT4, free thyroxine; CVD, cardiovascular disease; ASCVD, atherosclerotic cardiovascular disease; HF, heart failure. Small sample sizes in the hypothyroid (n=11) and hyperthyroid (n=5) subgroups may affect the stability of the estimates.

a) *p*<0.05.

**Table 3.** Effect of thyroid function on the increased 10-year CVD risk

Outcome	Total (n=246)		HbA1c <7% (n=112)		HbA1c ≥7% (n=134)	
	Adjusted coefficient (95% CI)	Adjusted *p*-value	Adjusted coefficient (95% CI)	Adjusted *p*-value	Adjusted coefficient (95% CI)	Adjusted *p*-value
CVD risk						
TSH	0.03 (–0.11 to 0.17)	0.683	0.17 (0.05 to 0.29)	0.005	–0.19 (–0.52 to 0.14)	0.262
fT4	0.93 (–5.03 to 6.89)	0.759	7.98 (0.53 to 15.43)	0.036	–2.93 (–13.58 to 7.73)	0.586
ASCVD risk						
TSH	0.03 (–0.05 to 0.11)	0.471	0.10 (0.02 to 0.18)	0.014	–0.07 (–0.26 to 0.12)	0.491
fT4	1.38 (–1.87 to 4.63)	0.402	5.02 (0.56 to 9.48)	0.028	–0.58 (–6.30 to 5.14)	0.841
HF risk						
TSH	–0.01 (–0.11 to 0.10)	0.920	0.12 (0.05 to 0.19)	0.001	–0.16 (–0.38 to 0.06)	0.142
fT4	–0.01 (–4.40 to 4.39)	0.998	5.79 (1.22 to 10.36)	0.014	–3.00 (–10.80 to 4.80)	0.446

CVD, cardiovascular disease; HbA1c, hemoglobin A1c; CI, confidence interval; TSH, thyroid-stimulating hormone; fT4, free thyroxine; ASCVD, atherosclerotic cardiovascular disease; HF, heart failure. Linear regression analysis was performed. Regression models were adjusted for HbA1c (among total participants), diabetes duration, antithyroid peroxidase antibody, and urinary iodine concentration. The subgroup analysis for patients with HbA1c <7% was exploratory. Given the cross-sectional design and the potential for multiple comparisons, these findings require cautious interpretation and further validation.

## Supplementary materials

Supplementary Tables 1 and 2 can be found at https://doi.org/10.12701/jyms.2026.43.28.

Supplementary Table 1. Baseline characteristics based on the 10-year CVD risk

Supplementary Table 2. Effect of thyroid function status on increasing 10-year CVD risk
