# Supplementary material for: Association between thyroid function and 10-year cardiovascular disease risk of patients with diabetes: a cross-sectional study using KNHANES 2013–2014
Source: J Yeungnam Med Sci. 2026 Apr 8;43:28. doi: 10.12701/jyms.2026.43.28 (PMC13373710; doi:10.12701/jyms.2026.43.28)
Supplement: Supplementary Table 1. — Baseline characteristics based on the 10-year CVD risk [file jyms-2026-43-28-Supplementary-Table-1.pdf]

Supplementary Table 1. Baseline characteristics based on the 10-year CVD risk

Characteristic	Total	10-Year CVD risk		*p*-value
		< 20% (Low-to-intermediate risk)	≥ 20% (High risk)	
No. of patients	246	198	48	
Sex				0.431
Male	144 (63.9)	108 (62.8)	36 (70.0)	
Female	102 (36.1)	90 (37.2)	12 (30.0)	
Age (yr)	54.3 ± 10.1	52.4 ± 9.7	64.6 ± 4.5	< 0.001
Smoking			0.611	
Former or never	175 (66.4)	145 (67.2)	30 (62.4)	
Current	71 (33.6)	53 (32.8)	18 (37.6)	
Drinking			0.196	
Never	29 (10.7)	23 (9.3)	6 (17.8)	
Ever	217 (89.3)	175 (90.7)	42 (82.2)	
Regular exercise	119 (48.4)	97 (49.1)	22 (44.6)	0.621
Hypertension	109 (37.7)	71 (29.3)	38 (81.3)	< 0.001
Dyslipidemia	60 (22.5)	53 (23.6)	7 (16.7)	0.501
Duration of diabetes (yr)	4.5 ± 6.0	3.8 ± 5.4	8.0 ± 7.5	< 0.001
Body mass index (kg/m^2)	25.8 ± 3.3	25.8 ± 3.5	25.5 ± 2.4	0.494
Waist circumference (cm)	87.6 ± 8.6	87.5 ± 8.9	88.2 ± 7.1	0.623
Systolic BP (mmHg)	123.5 ± 15.6	121.6 ± 14.4	133.0 ± 18.0	0.001
Diastolic BP (mmHg)	78.1 ± 10.8	78.5 ± 10.2	75.9 ± 13.3	0.281
Fasting glucose (mg/dL)	143.2 ± 37.7	143.3 ± 38.5	142.9 ± 34.1	0.950
HbA1c (%)	7.4 ± 1.5	7.3 ± 1.4	8.1 ± 1.5	0.002
Total cholesterol (mg/dL)	191.6 ± 36.1	192.7 ± 35.2	185.9 ± 40.5	0.364
HDL-cholesterol (mg/dL)	45.6 ± 10.4	46.1 ± 10.4	43.2 ± 9.8	0.078
LDL-cholesterol (mg/dL)	105.6 ± 36.3	106.0 ± 36.7	103.5 ± 34.9	0.689
Triglyceride (mg/dL)	201.6 ± 148.0	202.7 ± 142.7	195.9 ± 174.6	0.808
Creatinine (mg/dL)	0.9 ± 0.3	0.9 ± 0.2	1.1 ± 0.5	0.003
eGFR (mL/min/1.73 m^2)	83.8 ± 18.5	86.4 ± 16.3	70.1 ± 22.7	< 0.001
UACR (mg/g)	35.5 ± 109.9	19.1 ± 64.6	121.6 ± 213.2	0.006
UIC (μg/g)	537.1 ± 1,195.4	468.8 ± 912.3	890.6 ± 2,110.0	0.184
TSH (mIU/L)	3.2 ± 5.8	3.1 ± 4.0	3.9 ± 11.3	0.568
Free thyroxine (ng/dL)	1.2 ± 0.2	1.2 ± 0.2	1.2 ± 0.2	0.556
Anti-TPO antibody (IU/mL)	32.3 ± 165.8	26.5 ± 135.8	62.7 ± 273.8	0.411

Values are presented as number only, number (%), or mean ± standard deviation. All estimates were calculated by accounting for the complex survey design, including stratification, clustering, and sampling weights.

CVD, cardiovascular disease; BP, blood pressure; HbA1c, glycated hemoglobin; HDL, high-density lipoprotein; LDL, low-density lipoprotein; eGFR, estimated glomerular filtration rate; UACR, urinary albumin-creatinine ratio; UIC, urinary iodine/creatinine ratio; TSH, thyroid stimulating hormone; TPO, thyroid peroxidase.

https://doi.org/10.12701/jyms.2026.43.28
