# Supplementary material for: Association between thyroid function and 10-year cardiovascular disease risk of patients with diabetes: a cross-sectional study using KNHANES 2013–2014
Source: J Yeungnam Med Sci. 2026 Apr 8;43:28. doi: 10.12701/jyms.2026.43.28 (PMC13373710; doi:10.12701/jyms.2026.43.28)
Supplement: Supplementary Table 2. — Effect of thyroid function status on increasing 10-year CVD risk [file jyms-2026-43-28-Supplementary-Table-2.pdf]

Supplementary Table 2. Effect of thyroid function status on increased 10-year CVD risk

Thyroid function status	Total		HbA1c <7%		HbA1c ≥7%	
	Adjusted coefficient (95% CI)	Adjusted *p*-value	Adjusted coefficient (95% CI)	Adjusted *p*-value	Adjusted coefficient (95% CI)	Adjusted *p*-value
Euthyroid	1 (Reference)		1 (Reference)		1 (Reference)	
Hypothyroid	0 (–2.36 to 2.37)	>0.999	–1.1 (–4.35 to 2.15)	0.503	1.66 (–2.39 to 5.7)	0.417
Hyperthyroid	0.69 (–4.26 to 5.64)	0.783	4.09 (–3.97 to 12.14)	0.315	0.94 (–2.82 to 4.69)	0.620

CVD, cardiovascular disease; HbA1c, glycated hemoglobin; CI, confidence interval.
Linear logistic regression analysis was performed. Regression models were adjusted for HbA1c (among total participants), duration of diabetes, antithyroid peroxidase. antibody, and urinary iodine/creatinine ratio.
